# A pilot study to disentangle the infant gut microbiota composition and identification of bacteria correlates with high fat mass

**DOI:** 10.20517/mrr.2023.11

**Published:** 2023-06-25

**Authors:** Leonardo Mancabelli, Christian Milani, Federico Fontana, Nadia Liotto, Chiara Tabasso, Michela Perrone, Gabriele Andrea Lugli, Chiara Tarracchini, Giulia Alessandri, Alice Viappiani, Sergio Bernasconi, Paola Roggero, Fabio Mosca, Francesca Turroni, Marco Ventura

**Affiliations:** ^1^Department of Medicine and Surgery, University of Parma, Parma 43125, Italy.; ^2^Interdepartmental Research Centre “Microbiome Research Hub”, University of Parma, Parma 43124, Italy.; ^3^Laboratory of Probiogenomics, Department of Chemistry, Life Sciences and Environmental Sustainability, University of Parma, Parma 43124, Italy.; ^4^NICU, Fondazione IRCCS Ca’ Granda Ospedale Maggiore Policlinico, Milan 20122, Italy.; ^5^GenProbio srl, Parma 43124, Italy.

**Keywords:** Infant gut microbiota, microbiome, fat mass %, body weight

## Abstract

**Background:** At birth, the human intestine is colonized by a complex community of microorganisms known as gut microbiota. These complex microbial communities that inhabit the gut microbiota are thought to play a key role in maintaining host physiological homeostasis. For this reason, correct colonization of the gastrointestinal tract in the early stages of life could be fundamental for human health. Furthermore, alterations of the infant microbiota are correlated with the development of human inflammatory diseases and disorders. In this context, the possible relationships between intestinal microbiota and body composition during infancy are of great interest.

**Methods:** In this study, we have performed a pilot study based on 16S rRNA gene profiling and metagenomic approaches on repeatedly measured data on time involving a cohort of 41 Italian newborns, which is aimed to investigate the possible correlation between body fat mass percentage (FM%) and the infant gut microbiota composition.

**Results and conclusion:** The taxonomical analysis of the stool microbiota of each infant included in the cohort allowed the identification of a specific correlation between intestinal bacteria, such as *Bifidobacterium* and *Veillonella*, and the increase in FM%. Moreover, the analysis of the infant microbiome’s metabolic capabilities suggested that the intestinal microbiome functionally impacts the human host and its possible influence on host physiology.

## INTRODUCTION

The human gastrointestinal tract is colonized at birth by a variety of microbial communities that together will constitute the human gut microbiota^[1]^. It is now well established that these microbial communities play important roles in developing and maintaining the host’s physiology, contributing to the immunological, physiological, nutritional, and metabolic functionalities^[2-5]^. However, the human gut microbiota varies among individuals, and its composition is strongly related to the host genotype and environmental factors^[1,6]^. In this context, numerous studies concerning the infant’s microbiota have highlighted how different pre- and post-natal factors, such as delivery mode, gestational age, type of feeding and weaning, can influence infant microbiota composition^[1,7-11]^ and consequently its development^[12,13]^. C-section delivery, for example, is considered one of the most impacting events on the gut microbiota development, contributing to the colonization of the intestinal tract by bacteria derived from the environment and originating from the mother’s skin, which is thought to play a negative impact on the host health status in the mid-long term^[14,15]^.

Similarly, studies based on the analysis of preterm infants’ gut microbiota highlighted an alteration in the bacterial communities, characterized by a decrease of biodiversity, and its possible relation with life-threatening diseases, such as necrotizing enterocolitis (NEC)^[16,17]^ and late-onset sepsis^[18]^, and with the risk of developing inflammatory diseases, such as atopy, asthma, and obesity^[19-21]^. Moreover, the type of feeding and weaning has been related to the alteration of infant gut microbiota^[9,22,23]^. In detail, breastfed newborns have been demonstrated to promote a more stable and uniform population compared to formula-fed ones^[24]^.

Furthermore, several studies focused their interest on the association between the gut microbiota composition and the increase in body weight during the growth and development of infants, revealing a positive correlation with bacterial complexity^[25]^ and with bacterial genera, such as *Acinetobacter*, *Bifidobacterium*, *Collinsella*, *Enterococcus*, *Neisseria*, *Lactobacillus* and *Parabacteroides*^[26-28]^. Notably, studies focused on body weight gain in the first months of life are mainly based on body mass index (BMI), and only very few are directed at the most accurate measurement of body fat mass percentage (FM%)^[29,30]^. Therefore, to understand the possible relationships between body weight, FM%, and the infant gut microbiota composition, we have performed a pilot study based on repeatedly measured data on time from a cohort of 41 Italian newborns whose gut microbiota was investigated using 16S rRNA gene profiling and metagenomic approaches. In detail, the stool microbiota of each infant has been assessed through 16S rRNA gene profiling analysis at three different stages of life, i.e., at birth, at the fourth week, and at the thirteenth week, and correlated with the most relevant parameters of the subject, such as length of pregnancy, type of feeding, type of delivery, sex, body weight, and FM%. Furthermore, once the possible microbial markers correlated to the increase in FM% were identified, a metagenomic shotgun analysis was performed on a subset of samples to identify possible peculiar microbiome functional capabilities.

## METHODS

### Recruitment

In this pilot study, forty-one Italian infants from the NICU Fondazione IRCCS Ca’Granda Ospedale Policlinico of Milan were enrolled, trying to balance the clinical characteristics of the cohort of infants. In detail, all infants enrolled have not been hospitalized in Intensive Care. Moreover, the infants stayed at the nursery for a couple of days immediately after birth. In fact, all the infants included in the study are healthy with no severe pathologies, such as diseases related to the gastrointestinal and respiratory tract, and/or major comorbidities, and no antibiotic treatment. A single fecal sample was collected from each infant at three different time points, at birth (T0), i.e., a few days after birth, at the fourth week (T1), and at the thirteenth week (T2). These three time points were selected to study the evolution of microbiota in the early period of life in parallel to that of body composition. All three stool samples were collected in the onsite clinic.

The study protocol was approved by the Ethics Committee of the Fondazione IRCCS “Ca’Granda” Ospedale Policlinico of Milan (103_2016). Furthermore, written informed consent was obtained from the parents of all recruited infants.

### Data collection procedures

Infants were enrolled at birth. At enrolment, basic subject characteristics such as gestational age at birth, anthropometric parameters at each study point (body weight, length, and head circumference), and sex were recorded prospectively [Table 1 and Supplementary Table 1]. Gestational age was based on the last menstrual period and first-trimester ultrasonogram. The maternal characteristics were also collected [Supplementary Table 2].

**Table 1:** Clinical characteristics of the studied infant population

		***N* samples = 41**
**Sex**	Female	49%
	Male	51%
**Delivery type**	Term	68%
	Late preterm	32%
**Mode of delivery**	Vaginal	56%
	C-section	44%
**Feeding**	Exclusive breast milk	68%
	Breast milk and formula	29%
	Exclusively formula	2%

### Nutritional practices

Infants were fed on demand, and mothers were encouraged to breastfeed or express their milk according to their infant’s clinical condition. When human milk was unavailable or insufficient, formula feeding was started.

### Growth and body composition assessment

Anthropometric measurements (body weight, length, and head circumference) were assessed at birth (T0), at the fourth week (T1), and at the thirteenth week (T2). The weight of each baby was measured on an electronic scale accurate to 0.1 g (PEA POD Infant Body Composition System; COSMED, Italy). Body length was measured to the nearest 1 mm on a Harpenden neonatometer (Holtain, Crymych, UK). Furthermore, a non-stretch measuring tape measured the head circumference to the nearest 1 mm. All measurements were assessed by the trained medical staff of the author’s institution. Body composition was assessed using an air-displacement plethysmograph (PEA POD Infant Body Composition System; COSMED, Italy). A detailed description of the PEA POD’s physical design, operating principles, validation, and measurement procedures is provided elsewhere^[31]^. Briefly, the PEA POD assesses fat mass and fat-free mass by direct measurements of body mass and volume and the application of a classic densitometric model where the percentage of body fat is calculated using body density and pre-determined fat and fat-free mass density values. Body fat was defined as body weight minus fat-free mass. A constant fat mass density value of 0.9007 g/mL is used. Fat-free mass density values are calculated as the sum of the contribution of the various components in the fat-free mass compartment.

### Samples collection and DNA extraction

Fresh fecal samples were collected at birth (T0), at the fourth week (T1), and at the thirteenth week (T2), from infants born at the NICU Fondazione IRCCS Ca’ Granda Ospedale Policlinico of Milan and were immediately inactivated with DNA/RNA shield buffer (Zymo Research, USA) and subsequently submitted to the extraction of bacterial DNA using the protocol previously described^[32]^. The stool samples were collected by spontaneous evacuation and stored at room temperature in preservation tubes containing 2 mL of preservative and inactivating solution DNA/RNA shield buffer (Zymo Research, USA). Samples were then delivered to the laboratory of Probiogenomics, University of Parma, to perform the DNA extraction and sequencing. In detail, Bacterial DNA was extracted from fecal samples using the QIAamp Fast DNA Stool Mini kit following the manufacturer’s instructions (Qiagen Ltd., Strasse, Germany) and quantified using fluorometric Qubit quantification system (Life Technologies, Thermo Fisher Scientific, Waltham, Massachusetts, USA).

### 16S rRNA sequencing and profiling

Partial 16S rRNA gene sequences were amplified from extracted DNA using primer pair Probio_Uni and/Probio_Rev, targeting the V3 region of the 16S rRNA gene sequence^[33]^. 16S rRNA gene amplification and amplicon checks were carried out as previously described^[33]^. 16S rRNA gene sequencing was performed using a MiSeq (Illumina) according to the protocol previously reported^[33,34]^. Following sequencing, the FASTQ files were processed using a custom script based on the QIIME software suite^[35]^. Quality control-maintained sequences with a length between 140 and 400 bp and average sequence quality score of > 20, while sequences with homopolymers of > 7 bp and mismatched primers were omitted. 16S rRNA Amplicon sequence variants (ASVs) were defined at 100% sequence homology using DADA2, and ASVs represented by just a single sequence were removed. All reads were classified to the lowest possible taxonomic rank using QIIME 2, and a reference dataset from the SILVA database v.132.

#### Shotgun metagenomics sequencing

According to the manufacturer’s instructions, DNA library preparation was performed using the Nextera XT DNA sample preparation kit (Illumina, San Diego, CA, USA). First, 1 ng input DNA from each sample was used for the library preparation, which underwent fragmentation, adapter ligation, and amplification. Then, Illumina libraries were pooled equimolarly, denatured, and diluted to a concentration of 1.5 pM. Next, DNA sequencing was performed on a MiSeq instrument (Illumina) using a 2 × 250 bp Output sequencing Kit together with a deliberate spike-in of 1% PhiX control library.

#### Taxonomic classification of sequence reads

Taxonomic profiling of sequenced reads was performed employing the METAnnotatorX2 bioinformatics platform^[36,37]^. In detail, the downloaded FASTQ files were filtered to remove reads with a quality of  < 25, and to retain reads with a length of > 100 bp. Subsequently, a human host DNA filtering was performed through bowtie2 software^[38,39]^, following the METAnnotatorX2 manual^[37]^. Afterward, the taxonomic classification of 100,000 reads was achieved by means of MegaBLAST^[40]^ employing a manually curated and pre-processed database of genomes retrieved from the National Center for Biotechnology Information (NCBI), following the METAnnotatorX2 manual^[37]^.

#### Functional prediction

Functional profiling of the sequenced reads was performed with the METAnnotatorX2 bioinformatics platform^[36,37]^. In addition, functional classification of reads was performed to reveal metabolic pathways based on the MetaCyc database (release 24.1)^[41]^ through RAPSearch2 software^[42,43]^.

#### Statistical analysis

ORIGIN 2021 (https://www.originlab.com/2021) and SPSS software (www.ibm.com/software/it/analytics/spss/) were used to compute statistical analyses. Moreover, the similarities between samples (beta-diversity) were calculated by the Bray-Curtis dissimilarity matrix based on species abundance. Beta diversity was represented through Principal Coordinate Analysis (PCoA) using the function “ape” of the R suite package (http://www.rstudio.com/). Moreover, the available metadata and the various detected bacterial species were tested and plotted on the PCoA using the “envfit” and “plot” functions, respectively, in Rstudios (http://www.rstudio.com/). Permutational analysis of variance (PERMANOVA) analyses were performed using 999 permutations to estimate *P*-values for population differences in PCoA analyses. Furthermore, a correlation analysis between the available metadata and the various detected bacterial species of all samples at different time points was performed through Spearman’s rank correlation coefficient^[44]^. The False Discovery Rate (FDR) correction is applicated to all statistical analyses based on Benjamini and Hochberg correction^[45]^.

## RESULTS AND DISCUSSIONS

### Evaluation of stool microbiota inter-individual variability among subjects

**Figure 1:**
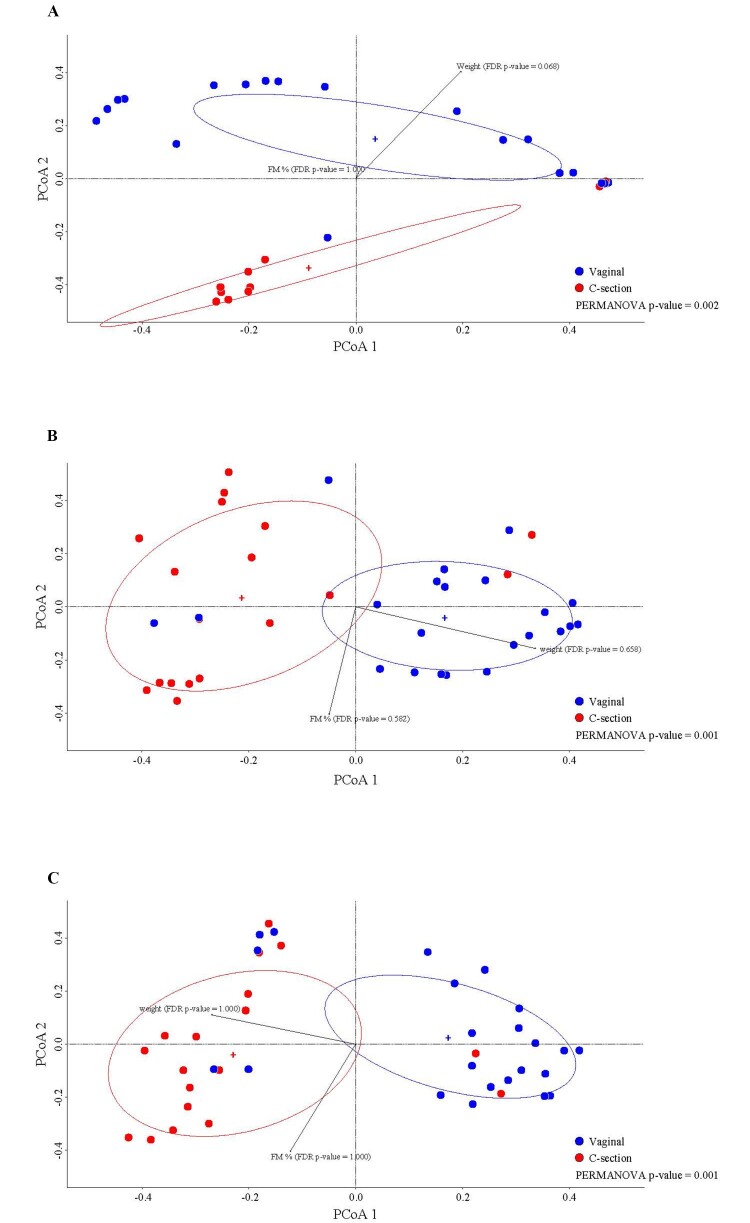
Evaluation of microbial beta-diversity**.** (A) displays the PCoA of the fecal samples at T0, subdivided by the type of delivery; (B) shows the PCoA of the fecal samples at T1, subdivided by the type of delivery; (C) reveals the PCoA of the fecal samples at T2, subdivided by the type of delivery. FDR: False discovery rate; PCoA: principal coordinate analysis; PERMANOVA: permutational analysis of variance.

In order to define the possible correlation/s between FM% and intestinal bacterial communities, a total of 123 fecal samples were collected from a heterogeneous infant population (*n* = 41) [Table 1]. The fecal microbiota of all stool samples collected in the framework of this pilot study was assessed by 16S rRNA gene profiling approach following the procedures reported previously^[33]^. The analysis of the FASTQ obtained from Illumina sequencing allowed to retrieve a total of 5,442,643 reads with an average per sample of 48,595 ± 12,976 after quality and human sequences filtering [Supplementary Table 3]. Unfortunately, 11 samples did not provide enough reads to obtain a reliable taxonomic profile, resulting in a final number of 112 analyzed samples, i.e., 31 samples at T0, 40 at T1, and 41 at T2 [Supplementary Table 3]. In order to identify possible population stratification, we have performed a beta-diversity analysis correlating the main parameters of the subject, such as duration of pregnancy, type of delivery, type of feeding, sex, body weight, and FM%, with the microbial genera that composed the gut microbiota. In detail, separate PCoA analyses at T0, T1, and T2 revealed a possible stratification of the samples at the different time points based on the type of delivery (PERMANOVA *P*-value < 0.05) [Figure 1A-C, Supplementary Table 4], suggesting an impact of the delivery mode on the development of the microbiota over time^[8,46]^. Moreover, PCoA analysis does not appear to show further stratification based on the other subject parameters, such as duration of pregnancy, type of feeding, sex, body weight, and FM%.

Inspection of the predicted taxonomic profiles showed that the infant cohort is mainly characterized by the genera *Streptococcus* (average 4.11% ± 7.70%, prevalence 97%), *Bacteroides* (average 19.66% ± 24.70%, prevalence 96%), *Bifidobacterium* (average 7.41% ± 11.75%, prevalence 91%) and *Escherichia-Shigella* (average 23.41% ± 29.13%, prevalence 88%) [Supplementary Table 5], bacteria typical of the gastrointestinal tract^[9,47]^. Moreover, a specific descriptive taxonomical comparison between the groups of natural and C-section-delivered infants revealed possible differences in the microbiota composition. Specifically, focusing on the bacterial taxa with a total prevalence > 50 % [Table 2], *Escherichia-Shigella*, *Bacteroides,* and *Parabacteroides* genera seemed to be associated and to be persistent in vaginal delivery samples. In contrast, *Streptococcus*, *Flavobacterium*, *Lactobacillus*, *Staphylococcus*, *Clostridium* sensu stricto 1, *Rothia,* and *Enterococcus* genera resulted related to C-section delivery [Table 2]. These results are in agreement with previous studies that indicated *Bacteroides* and *Escherichia-Shigella* as two of the dominant bacterial genera of vaginally delivered infants’ gut microbiota^[9,47,48]^. Furthermore, the decrease in babies born by C-section of *Bacteroides* genus in favor of environmental bacteria, such as *Streptococcus*, *Staphylococcus,* and *Clostridium*, could be related to external contamination during childbirth and a consequent increased competition within the bacteria of the infant’s gut^[49]^. Moreover, in order to identify the possible correlation between the type of delivery and the other variables, such as duration of pregnancy and sex, a cross-tabulation analysis was performed, highlighting the independence of these variables [Supplementary Table 6].

**Table 2:** Bacterial taxa with a total prevalence > 50% and persistent higher abundance in vaginal or C-section delivery samples

		**T0**					**T1**					**T2**				
		**Average**		**Standard deviation**		**Higher abundance in**	**Average**		**Standard deviation**		**Higher abundance in**	**Average**		**Standard deviation**		**Higher abundance in**
**Taxonomy**	**Total prevalence**	**Vaginal**	**C-section**	**Vaginal**	**C-section**		**Vaginal**	**C-section**	**Vaginal**	**C-section**		**Vaginal**	**C-section**	**Vaginal**	**C-section**	
*Bacteroides*	95.54%	34.53%	1.19%	32.35%	1.34%	Vaginal	23.36%	3.58%	19.63%	9.98%	Vaginal	34.51%	5.16%	22.73%	14.83%	Vaginal
*Escherichia-Shigella*	87.50%	47.02%	19.98%	39.63%	40.23%	Vaginal	23.86%	6.39%	23.11%	14.30%	Vaginal	22.96%	14.82%	18.31%	21.39%	Vaginal
*Parabacteroides*	54.46%	3.84%	0.38%	5.96%	0.76%	Vaginal	5.01%	2.54%	7.99%	10.73%	Vaginal	3.54%	0.53%	5.28%	2.22%	Vaginal
*Clostridium* sensu stricto 1	59.82%	0.04%	10.36%	0.12%	21.94%	C-section	3.78%	13.68%	10.76%	18.17%	C-section	3.25%	10.62%	7.84%	16.68%	C-section
*Enterococcus*	57.14%	0.07%	0.41%	0.19%	0.83%	C-section	0.17%	0.84%	0.50%	2.54%	C-section	0.08%	0.30%	0.14%	0.61%	C-section
*Flavobacterium*	57.14%	0.04%	0.29%	0.08%	0.38%	C-section	0.01%	0.06%	0.02%	0.14%	C-section	0.01%	0.02%	0.01%	0.03%	C-section
*Lactobacillus*	59.82%	0.04%	0.17%	0.08%	0.20%	C-section	0.06%	0.39%	0.14%	1.11%	C-section	0.48%	1.47%	1.21%	5.73%	C-section
*Rothia*	71.43%	0.06%	0.49%	0.14%	0.90%	C-section	0.30%	0.82%	0.36%	2.03%	C-section	0.08%	0.16%	0.19%	0.28%	C-section
*Staphylococcus*	59.82%	0.07%	16.67%	0.18%	31.71%	C-section	0.27%	1.97%	0.52%	6.35%	C-section	0.03%	0.47%	0.05%	1.54%	C-section
*Streptococcus*	97.32%	1.39%	9.71%	4.38%	16.44%	C-section	4.16%	6.31%	5.36%	7.53%	C-section	2.72%	3.65%	6.30%	6.51%	C-section

### Identification of possible correlation between infant gut microbiota and fat mass

The possible correlation and impact of the gut microbiota composition on the host’s body weight and BMI have been extensively investigated^[50-55]^. Nevertheless, few studies have investigated the relationship between microbiota and FM% in children and adults^[29,30,53]^. In this context, the metadata collected in this study enabled a specific correlation analysis between infant FM%, body weight, and intestinal microbiota during the first stage of life [Supplementary Table 1]. In detail, the correlation analysis of all samples at different time points suggested that four bacterial taxa were significantly (*P*-value < 0.05) positively correlated to the infant’s body weight and FM%. In contrast, three bacterial genera were significantly negatively correlated [Table 3]. Interestingly, the *Bifidobacterium* genus appears to be related to an increase in body weight and fat mass, suggesting their positive key role in newborn growth that, together with other already well-known functional roles exploited by this microbial group on the human body, reinforces the notion of their importance as a crucial bacterial genus for the foundation of human health^[56,57]^. Furthermore, among the bacterial taxa identified to exploit a possible role in the modulation of body weight and fat mass, *Bifidobacterium*, *Veillonella,* and *Klebsiella* genera have already been previously described as taxa characteristic and dominant of the infant gut microbiota, also referred to as infant community state types (ICSTs)^[9,47]^. These results may suggest the importance of the microbiota composition in the first months of life and its consequent effect on the absorption and assimilation of nutrients/fat and the physiology and health of the host.

**Table 3:** Correlation between bacterial taxa and weight or FM%. Only significant statistical correlations are reported

	**Weight**		**FM (%)**	
**Taxonomy**	**Spearman’s rank correlation coefficient**	**FDR *P*-value** **(Benjamini and Hochberg correction)**	**Spearman’s rank correlation coefficient**	**FDR *P*-value (Benjamini and Hochberg correction)**
*Bifidobacterium*	0.339	0.0023	0.362	0.0009
*Veillonella*	0.446	0	0.416	0.0001
*Idiomarina*	0.253	0.0435	0.26	0.0361
*Klebsiella*	0.263	0.0328	0.354	0.0012
*Haemophilus*	0.442	0	0.358	0.0011
*Alistipes*	-0.291	0.0134	-0.26	0.0353
*Phascolarctobacterium*	-0.282	0.0179	-0.25	0.0474

FDR: False discovery rate; FM: fat mass.

### Preliminary evaluation of the functionality of gut microbiome related to FM%

**Figure 2:**
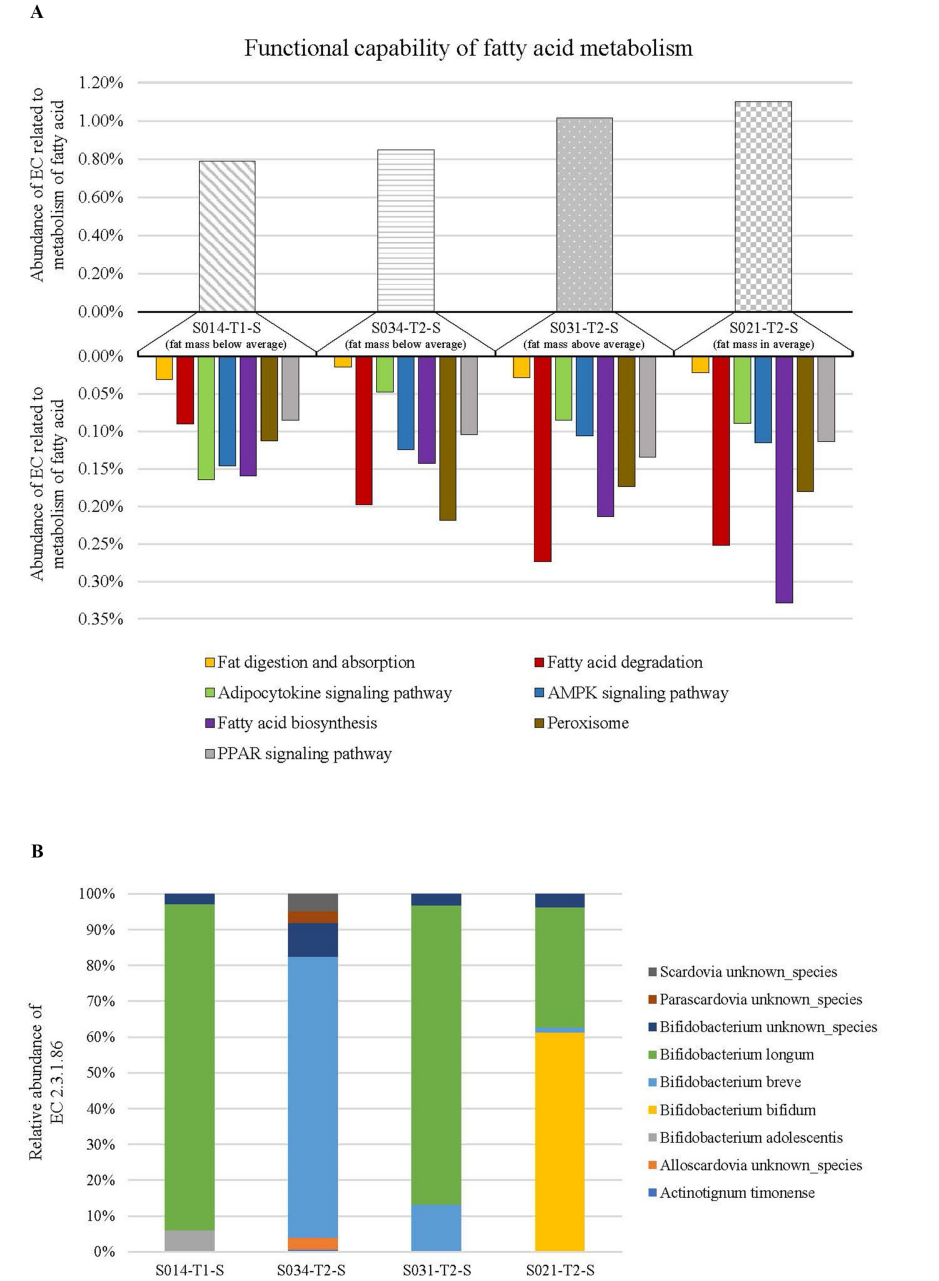
Functional capability of fatty acid metabolism. (A) shows a bar plot reporting the percentage abundance of EC enzyme related to each selected sample’s metabolism of fatty acid; (B) indicates the percentage of microorganisms presenting genes related to EC 2.3.1.86 in each sample. EC: Enzyme commission; EC 2.3.1.86: fatty-acyl-CoA synthase system.

The 16S rRNA gene profiling analysis performed in this study allowed to establish a correlation between body weight and FM% and specific intestinal bacteria [Table 3]. In order to identify the possible metabolic role exploited by these microorganisms on the FM% and body weight of the host, a preliminary shotgun metagenomic analysis was performed. In detail, a preliminary in-depth metagenomic analysis was achieved by selecting a subset of four samples based on their FM% and gut microbiota composition. In particular, samples with a high abundance (> 20%) of at least one of the taxa previously observed positively or negatively correlated with FM% and body weight were selected [Table 3 and Supplementary Table 5]. Therefore, four samples were selected, i.e., S031-T2, S014-T1, S034-T2, and S021-T2, re-named S031-T2-S, S014-T1-S, S034-T2-S, and S021-T2-S, respectively. In detail, sample S031-T2-S, C-section delivered and exclusively breastfed, had a high abundance of bifidobacteria (50.86%) and fat mass % above-average (32.2 compared to the T2 average of 25 ± 5). Furthermore, sample S014-T1-S, vaginally delivered and exclusively breastfed, was characterized by a high abundance of *Parabacteroides* genus (22.25%) and fat mass % below-average (10.2 compared to the T1 average of 16 ± 5). Thus, samples S034-T2-S, vaginally delivered and exclusively breastfed, and S021-T2-S, C-section delivered and exclusively breastfed, had a high abundance of *Veillonella* genus (37.85% and 26.05%, respectively), but S034-T2-S had a fat mass % below-average (11.2 compared to the T2 average of 25 ± 5) while S021-T2-S had fat mass % in the average (27.3 compared to the T2 average of 25 ± 5). In order to explore the metabolic capabilities of the microbiome of each selected sample, a screening of metabolic enzymatic reactions based on the MetaCyc database^[41]^ and the Enzyme Commission (EC) classification was performed. In particular, we focused on the EC involved in fatty acid metabolism to investigate the possible correlation between the composition of the intestinal microbiome and FM% [Figure 2A]. Interestingly, a specific descriptive comparison between samples revealed that subjects with a high FM%, i.e., S031-T2-S and S021-T2-S samples, displayed a greater number of reads associated with fatty acid metabolism than infants with low FM%, i.e., S014-T1-S and S034-T2-S samples [Figure 2A], suggesting a possible correlation between individual’s fat mass percentage and microbiome composition. In detail, samples S031-T2-S and S021-T2-S showed a larger number of enzymes involved in fatty acid biosynthesis compared to the two samples with low FM% [Supplementary Table 7], mainly represented by fatty-acyl-CoA synthase system (EC 2.3.1.86), indicating an increased capacity for fatty acid synthesis. Moreover, a specific investigation through METAnnotatorX2 software^[37,58]^ was performed to identify which bacterial species of the different samples possessed genes predicted to encode the EC 2.3.1.86 [Figure 2B]. Remarkably, the analysis revealed that this EC enzyme was encoded in all samples by genes mainly presented in microorganisms belonging to the *Bifidobacterium* genus, such as *B. longum and B. breve* species, suggesting a possible impact of this bacterial taxon on FM% in relation to its abundance [Figure 2B]. These preliminary results could confirm the notion that host physiology is closely correlated to the composition of the intestinal microbiome, suggesting the importance of proper bacterial colonization to maintain infant health. In particular, these results could support the hypothesis that the correct colonization of the gastrointestinal tract in the first months of life plays a key role in developing and maintaining the host’s state of health in the short and long term^[12,59,60]^.

In conclusion, the human gut microbiota has been increasingly recognized as playing a role in human health and disease. Recent studies have suggested that gut microbiota may be involved in regulating human body weight and fat mass. In the current study, we have investigated the potential correlation between the infant gut microbiota and FM%, also considering the main parameters of the subject, such as duration of pregnancy, type of feeding, type of delivery, sex, and body weight. Interestingly, the analysis of the most relevant subject parameters allowed to identify possible differences in microbiota composition based on the type of delivery. In detail, vaginal delivery samples were characterized by the presence of *Escherichia-Shigella*, *Bacteroides,* and *Parabacteroides* genera, while C-section delivery samples seemed related to environmental bacteria probably obtained during childbirth. Moreover, the 16S rRNA gene profiling analysis allowed to correlate certain bacterial taxa with increased or decreased body weight and fat mass. Remarkably, some of the bacterial genera identified to exploit a possible role in the modulation of body weight and fat mass are commonly found in the gut microbiota of infants, indicating the significance of early-life gut microbiome composition on nutrient and fat absorption. Furthermore, a prediction of the microbiome metabolic functionality of a subset of infant samples revealed differences in the metabolic capabilities related to fatty acids. Specifically, samples with a higher fat mass % showed a higher number of enzymes involved in fatty acid biosynthesis, indicating the infant gut microbiota’s possible role in the host’s body weight and fat mass. Moreover, this analysis revealed that the *Bifidobacterium* genus represents the main bacterial taxon providing a genetic repertoire involved in fatty acid biosynthesis. Thus, this pilot study confirms the potential correlation between the infant gut microbiome and fat mass development. Further research is needed to fully understand the molecular mechanisms behind this intriguing relationship and determine whether gut microbiome manipulation may be a useful strategy for managing an individual’s body weight/fat mass. In fact, several studies reported that rapid weight gain in the first months of life could be an important risk factor for overweight and/or obesity and subsequent predisposition to an unfavorable metabolic profile in early adulthood^[61-63]^, suggesting the first months of life as a critical window for adiposity programming. Moreover, further longitudinal investigations regarding the type of feeding in early life could allow to identify/clarify the possible relationship between microbiota, feeding, and fat mass. Thus, identifying specific bacterial biomarkers, such as some bifidobacterial species, can contribute to establishing novel strategies able to influence the individual’s body weight and fat mass through the modulation of the microbiota composition.

## DECLARATIONS

### Availability of data and materials

Raw sequences of the Italian 16S rRNA gene and shallow-shotgun sequencing data are accessible through SRA under study accession (NO. PRJNA930371).

### Copyright

© The Author(s) 2023.
